# Evaluation of lateral flow devices for postmortem rabies diagnosis in animals in the Philippines: a multicenter study

**DOI:** 10.1128/jcm.00842-23

**Published:** 2023-11-22

**Authors:** Jeffrey L. Cruz, Alyssa M. Garcia, Nobuo Saito, Maria Glofezita O. Lagayan, Rainelda C. Dela Peña, Michael S. Usana, Steciousneil P. Agustin, Judith Z. Tattao, Christine V. Mamauag, Ofelia P. Ducayag, Heather Laxe F. Nabus, Princess Diana D. L. Flores, Ralph Joselle A. Fabon, Rogelio P. Peñaflor, Dave Christopher G. Viñas, Carla A. Limsan, Rona P. Bernales, Maria Erlinda T. Llames, Lerisa E. Balopeños, Ramir G. Morales, Ana Marie Cristina V. Migriño, Orfel June S. Calunsag, Josephine J. Datoy, Ken Y. Palma, Maria Corazon B. Sepulveda, Ma Noreen J. Eng, Jobienaur S. Moscoso, Sheena Mae F. Julabar, Jaira D. Mauhay, Mark Joseph M. Espino, Christine Joyce M. Javier, Kazunori Kimitsuki, Akira Nishizono

**Affiliations:** 1 Department of Agriculture, Bureau of Animal Industry, Quezon, Philippines; 2 Department of Microbiology, Faculty of Medicine, Oita University, Yufu, Oita, Japan; 3 Regional Animal Disease Diagnostic Laboratory I, Sta Barbara, Pangasinan, Philippines; 4 Regional Animal Disease Diagnostic Laboratory II, Tuguegarao, Cagayan, Philippines; 5 Regional Animal Disease Diagnostic Laboratory CAR, Baguio, Benguet, Philippines; 6 Regional Animal Disease Diagnostic Laboratory IVA, Lipa, Batangas, Philippines; 7 Regional Animal Disease Diagnostic Laboratory IVB, Naujan, Oriental Mindoro, Philippines; 8 Regional Animal Disease Diagnostic Laboratory IVB - Satellite Laboratory, Puerto Princesa, Palawan, Philippines; 9 Regional Animal Disease Diagnostic Laboratory V, Pili, Camarines Sur, Philippines; 10 Regional Animal Disease Diagnostic Laboratory VI, Iloilo, Philippines; 11 Regional Animal Disease Diagnostic Laboratory VII, Mandaue, Cebu, Philippines; 12 Regional Animal Disease Diagnostic Laboratory IX, Zamboanga, Zamboanga del Sur, Philippines; 13 Davao City Animal Disease Diagnostic Laboratory, Davao, Davao del Sur, Philippines; 14 Regional Animal Disease Diagnostic Laboratory XII, General Santos, South Cotabato, Philippines; 15 Research Center for Global and Local Infectious Diseases, Oita University, Yufu, Oita, Japan; Boston Children's Hospital, Boston, Massachusetts, USA

**Keywords:** rabies, lateral flow devices, the Philippines, multicenter evaluation

## Abstract

Expansion of the use of lateral flow devices (LFD) for animal rabies diagnosis can help mitigate the widespread underreporting of rabies. However, this has been hindered by the limited number and small sample size of previous studies. To overcome this limitation, we conducted a multicenter study with a larger sample size to assess the diagnostic accuracy of the ADTEC LFD for postmortem rabies diagnosis in animals. Thirteen governmental animal diagnostic laboratories in the Philippines were involved in this study, and 791 animals suspected of having rabies were tested using both the direct fluorescence antibody test (DFAT) and ADTEC LFD between August 2021 and October 2022. The LFD demonstrated a sensitivity of 96.3% [95% confidence interval (CI): 94.1%–97.9%] and a specificity of 99.7% (95% CI: 98.4%–100%). Notably, false-negative results were more likely to occur in laboratories with lower annual processing volumes of rabies samples in the previous years (adjusted odds ratio 4.97, 95% CI: 1.49–16.53). In this multicenter study, the high sensitivity and specificity of the LFD for the diagnosis of animal rabies, compared to that of the DFAT, was demonstrated, yet concerns regarding false-negative results remain. In areas with limited experience in processing rabies samples, it is essential to provide comprehensive training and careful attention during implementation.

## INTRODUCTION

Despite being a vaccine-preventable disease, rabies remains a substantial public health concern in many rabies-endemic countries (1 – 3). Most human rabies deaths are caused by dog bites in Africa and Asia (4). Controlling canine rabies is the most effective strategy for mitigating the global burden of human rabies (5). Establishing robust surveillance systems for detecting animal rabies is a crucial component for controlling rabies that can clarify the disease burden and monitor control measures such as mass dog vaccination (6). The laboratory confirmation of rabies in animals necessitates an examination of brain samples of deceased animals or the identification of clinical signs. The direct fluorescent antibody test (DFAT) serves as the gold standard test in most endemic countries (7, 8) and is recommended by global organizations such as the World Health Organization (WHO) and the World Organization for Animal Health (WOAH), alongside other tests, including the direct rapid immunohistochemical test (DRIT) and reverse transcription PCR (RT-PCR) (7, 9, 10). The DFAT relies on the detection of viral antigens in brain impressions stained with fluorophore-conjugated antibodies using fluorescence microscopy (9). The limitations of DFAT include the need for an expensive fluorescent microscope, high-quality antibody conjugates, and skilled technicians capable of distinguishing positive rabies antigens from non-specific fluorescence. DRIT, which eliminates the need for fluorescent microscopes, has been established as a simpler alternative to DFAT (11, 12). However, it still requires a light microscope and a cold chain to store anti-rabies antibodies, and it is not commercially available. Although various RT-PCR methods have been developed, they still require specialized equipment and are difficult to implement in most endemic areas (9, 10, 13). Lateral flow devices (LFDs) have been developed to rapidly detect rabies virus antigens. LFDs offer notable advantages, such as rapidity, ease of use, and lack of additional equipment. These tests are particularly valuable in situations where the reference test (DFAT) is unavailable. A critical concern associated with the rabies LFD test is its lower sensitivity than the DFAT test, with variations in sensitivity observed among commercially available kits. Certain kits exhibit unacceptably low diagnostic accuracy (8, 14, 15). The Bionote LFD kit has been extensively evaluated and has exhibited high sensitivity in previous studies (15 – 23). ADTEC LFD has been assessed in multiple countries, including the Philippines, Sri Lanka, Bhutan, and Thailand, demonstrating high sensitivity and specificity (8, 24 – 26). A study conducted in the Philippines showed high sensitivity of the Bionote kit 95%; [95% confidence interval (CI), 88%–98%] and the ADTEC kit (94%; 95% CI, 87%–97%), while the Elabscience kit displayed 0% sensitivity. Moreover, when brain samples were collected using the simplified sampling method (straw sampling method), a high sensitivity of 94% (95% CI, 84%–99%) was observed, and thus, this can be used in resource-limited settings (16, 26).

Although LFD is simpler and faster than DFAT, it has not been widely adopted in most endemic countries. One contributing factor is insufficient evidence regarding the diagnostic accuracy of LFD in endemic areas, as most previous studies were relatively small-scale (8, 14 – 28). A single-center study in the Philippines has demonstrated the high diagnostic accuracy of ADTEC LFD (8, 26). However, the study was conducted by well-trained technicians in a highly rabies-endemic area, raising concerns about the generalizability of the results to other regions.

We conducted a large-scale multicenter study in a rabies-endemic country. Our primary objectives were to evaluate the diagnostic accuracy of LFD for diagnosing animal rabies and to explore the potential factors associated with the discrepancy between LFD and DFAT. This study represents the first large multicenter evaluation of LFD for rabies diagnosis in a rabies-endemic country to provide robust evidence to the field.

## MATERIALS AND METHODS

### Participating laboratories

**Fig 1:**
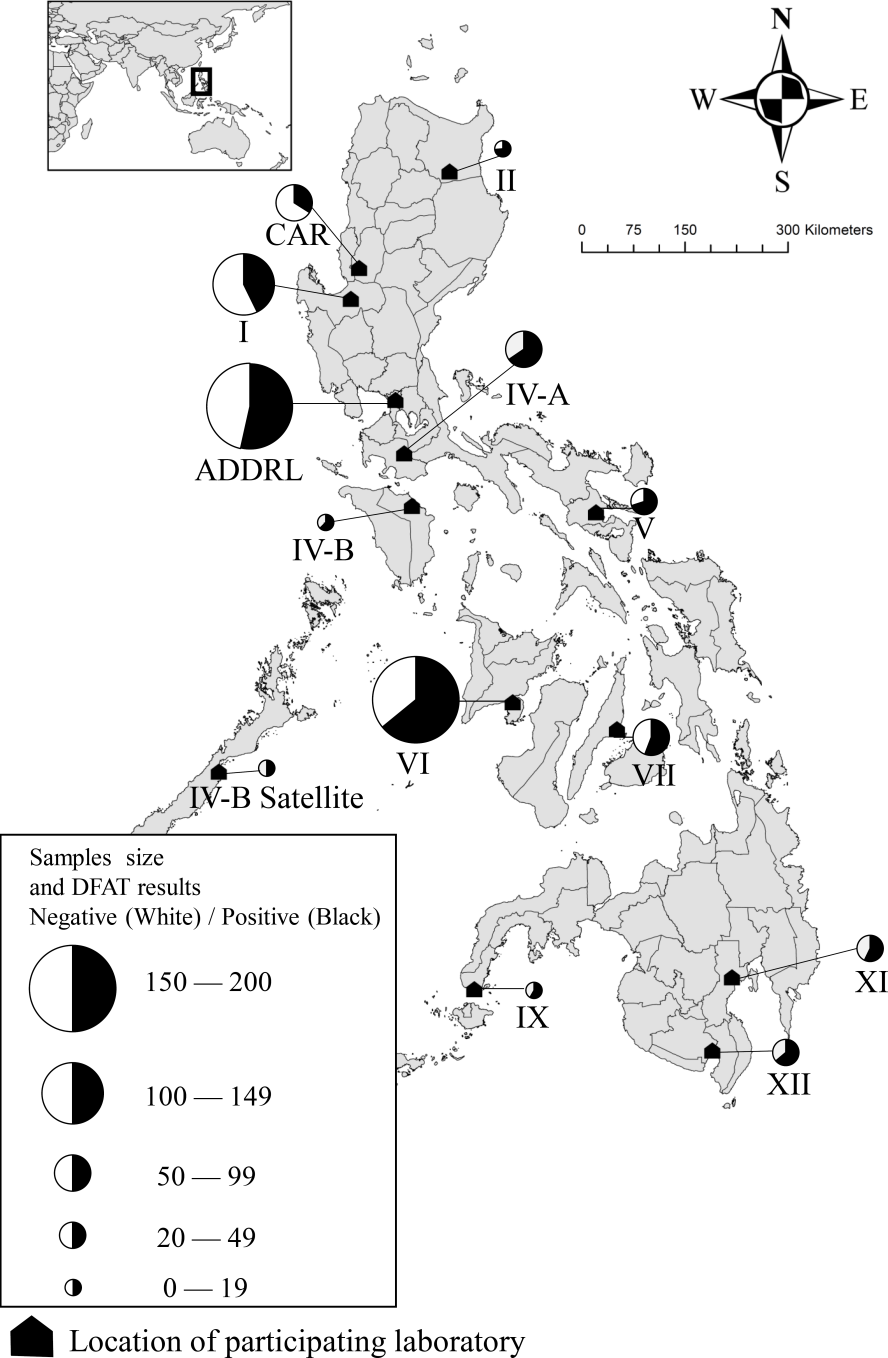
Locations of participating laboratories and sample sizes with direct fluorescent antibody test results.

The study protocol was reviewed by the research review board of the Bureau of Animal Industry in the Philippines. Animal ethical approval was waived because only brain samples from animals with suspected rabies with carcasses or animal heads were submitted to the laboratories for rabies testing. This multicenter study was conducted to assess the diagnostic accuracy of LFD for the postmortem diagnosis of animal rabies, with DFAT serving as the reference test. The study period was between 1 August 2021 and 31 October 2022. In August 2021, 19 government laboratories conducted animal rabies tests using DFAT. In 2018, the postmortem diagnostic testing of rabies using DFAT was conducted on 3,997 animals in the Philippines, of which 1,227 were found to be positive (29). Three laboratories (Research Institute of Tropical Medicine, Regional Animal Laboratory III, and Regional Animal Laboratory III satellite) were excluded because the ADTEC LFD had previously been evaluated in these facilities (8, 26). After excluding those 3 laboratories, 16 were invited to participate in the study. Between November 2021 and April 2021, LFD kits were distributed to these 16 laboratories, followed by online training in sample preparation and use of LFDs (8, 26). Of the 16 invited laboratories, 13 participated in this study (Fig. 1). The primary reasons for the lack of participation of the three laboratories were the limited number of rabies samples during the study period and their inability to allocate resources due to the occurrence of outbreaks of other infectious diseases, such as African swine fever and avian influenza. We included the Davao City Veterinary Office in the study because the laboratory performs rabies testing in the region instead of the regional animal laboratory IX.

### Baseline survey

We initially conducted an online survey to determine the status of rabies testing in participating laboratories from 2019 to 2020 (see Table S1 in the supplemental material). The survey showed that 23–262 samples per laboratory are tested annually for DFAT. Among them, three laboratories processed fewer than 50 samples per year, whereas four laboratories processed more than 200 specimens. Three laboratories had previous experience with LFD tests for rabies. Veterinarians primarily performed DFAT, although non-veterinarians, such as medical technicians, also conducted the tests in some laboratories.

### Study procedure

The participating laboratories routinely accept decapitated animal heads and offer DFAT as a complimentary rabies confirmatory test to both government agencies and private individuals. This is implemented as part of a national passive surveillance system (29). In this study, we instructed the laboratories to perform LFD tests for research purposes alongside their routine DFAT. We included the results of this study when the participating laboratories performed both DFAT and LFD tests. The study team created a standardized sample report form to document the characteristics of rabies-suspected animals and the test results. Laboratories were requested to submit a sample report form along with photographs of the LFD results. When rabies-suspected animals were submitted to the laboratory, both LFD and DFAT were performed. This study did not standardize the test order, depending on each laboratory’s decision. In this study, laboratories were permitted to perform the LFD test after briefly storing samples in a −20°C freezer if they could not carry out the LFD on the same day as the DFAT. As long-term storage can reduce the sensitivity of LFDs, we excluded samples that had been stored for more than 90 days (14, 15, 25, 30). In this study, blinded comparisons between the two tests were not employed to prevent examiner bias.

### Sampling and LFD procedure

The laboratory staff obtained brain tissue samples by either opening the skull or by a simplified sampling method using drinking straws (occipital foramen route brain sampling without opening the skull). The baseline survey revealed that only two laboratories had previously used a simplified sampling method. For this study, we used the Rabies Ag test (ADTEC Co., Ltd., Oita, Japan; lot nos. 2102, 2103, 2104, and 2201). Therefore, we recommend using the brainstem as the sample for LFD. The brain tissue was diluted with the assay buffer and homogenized using the sample Masher kits included in the ADTEC LFD kits. The supernatants were added to the sample hole in the cassette and the results were read after 15 min. The final results were observed as red-colored bands in the test and control zones (see Fig. S1) (24). The detailed methodology of the LFD has been described previously (8, 26). As we did not standardize the DFAT method for the study, each participating laboratory performed DFAT according to their own manuals. In the manual procedure in the Philippines, it is recommended to use the hippocampus, cerebellum, and brainstem for DFAT (29). Whereas we examined factors contributing to discrepancies between the DFAT and LFD results, we did not conduct additional confirmatory tests, such as RT-PCR, for samples showing discrepancies. When laboratories received samples, laboratory technicians assessed the sample conditions and defined them as acceptable or unacceptable for the DFAT. For the acceptable condition, no signs of liquefaction or significant degradation are present in the tissues, and they should exhibit a natural pinkish-gray color without any signs of discoloration. For the unacceptable condition, the sample emits a putrid or foul smell, showing liquefaction or any greenish or unnatural discoloration.

### User experience

We conducted a user experience survey between 15 July 2022, and 16 January 2023. After the laboratory staff performed a certain number of LFD tests, we requested them to answer online questionnaires and then conducted qualitative interviews online to explore the usability of LFD and gather opinions.

### Data management and analysis

The research team extracted data from the report forms and entered them in Google Forms. The data were converted into Microsoft Excel 2019 (Microsoft Corporation, Redmond, WA, USA). Data analysis was performed using the Stata Statistical Software, version 15.1 (StataCorp, College Station, TX, USA). The sensitivity and specificity of LFD, along with 95% CIs, were determined and compared with those of the DFAT, which served as a reference test (29). Categorical variables were used to categorize laboratory groups based on the yearly number of DFAT tests in 2019 and 2020 (≥200, 100–199, and <100). Fisher’s exact test was used to identify statistical differences among categorical variables. Statistical significance was set at *P* < 0.05. We used a multivariate logistic regression model to examine the factors associated with the discrepancy in results and estimate the adjusted odds ratios (AOR). The final model included any variables with a *P*-value <0.05, as determined by univariate analysis. The reporting of the study followed the guidelines outlined in the STARD (Strengthening the Reporting of Observational Studies in Epidemiology) statement (see Table S2 in the supplemental material).

## RESULTS

Between August 2021 and October 2022, 791 results were included in the study. Thirteen results were excluded because of unavailable DFAT results or samples stored for more than 90 days prior to LFD testing. Therefore, 778 results were included in the analysis (Table 1; Table S2 ). Most of the animals were dogs (*n* = 682, 87.7%), followed by cats (*n* = 77, 9.9%), and the DFAT positivity was higher for dogs than for cats. Of these animals, 41.8% were aged less than 12 months old (Table 1). The number of test results per laboratory varied from 6 to 172 tests. While three laboratories submitted more than 100 results, six laboratories provided fewer than 30 results. The positivity rates for DFAT in laboratories range from 30% to 70%. Among the LFD tests, 58.2% (*n* = 453) were performed within 6 days of receiving the samples, whereas 37.8% (*n* = 294) were performed between 7 and 90 days. A total of 21.1% of the brain specimens were collected by a straw sampling method. Five LFD lots were used in this study. While only four samples were applied to lot no. 2012, around 200 kits of other LFD lots were used (nos. 2102, 2103, 2104, and 2201). Of the 778 submitted samples, 434 tested positive for DFAT, and 418 tested positive for LFD. The sensitivity and specificity of LFD were 96.3% (95% CI: 94.1%–97.9%) and 99.7% (95% CI: 98.4%–100%), respectively (Table 2).

**TABLE 1:** Characteristics of samples from animals suspected of having rabies submitted to participating laboratories according to direct fluorescent antibody test results^*b*^

	Total, N (%) (*n* = 778)	DFAT negative, N (%) (*n* = 344)	DFAT positive, N (%) (*n* = 434)	Positivity (%)
Species				
Dog	682 (87.7)	267 (77.6)	415 (95.6)	60.9
Cat	77 (9.9)	70 (20.3)	7 (1.6)	9.1
Other^*a*^	10 (1.3)	2 (0.6)	8 (1.8)	80.0
Unknown or missing value	9 (1.2)	5 (1.5)	4 (0.9)	44.4
Age				
<3 months	98 (12.6)	66 (19.2)	32 (7.4)	32.7
4–11 months	227 (29.2)	121 (35.2)	106 (24.4)	46.7
1–2 years	205 (26.3)	71 (20.6)	134 (30.9)	65.4
>=3 years	119 (15.3)	43 (12.5)	76 (17.5)	63.9
Unknown or missing value	129 (16.6)	43 (12.5)	86 (19.8)	66.7
Laboratory				
ADDRL	172 (22.1)	80 (23.3)	92 (21.2)	53.5
CAR	50 (6.4)	33 (9.6)	17 (3.9)	34.0
I	131 (16.8)	75 (21.8)	56 (12.9)	42.7
II	19 (2.4)	5 (1.5)	14 (3.2)	73.7
IV-A	84 (10.8)	29 (8.4)	55 (21.7)	65.5
IV-B	8 (1.0)	3 (0.9)	5 (1.2)	62.5
IV-B_satellite	6 (0.8)	3 (0.9)	3 (0.7)	50.0
V	20 (2.6)	6 (1.7)	14 (3.2)	70.0
VI	167 (21.5)	60 (17.4)	107 (24.7)	64.1
VII	52 (6.7)	23 (6.7)	29 (6.7)	55.8
IX	12 (1.5)	5 (1.5)	7 (1.6)	58.3
Davao city (region XI)	21 (2.7)	9 (2.6)	12 (2.8)	57.1
XII	36 (4.6)	13 (3.8)	23 (5.3)	63.9
Duration between date of sample received and LFD test (Days)				
Same day	155 (19.9)	59 (17.2)	96 (22.1)	61.9
1–2 days	181 (23.3)	71 (20.6)	110 (25.3)	60.8
3–6 days	117 (15.0)	54 (15.7)	63 (14.5)	53.8
7–30 days	242 (31.1)	121 (35.2)	121 (27.9)	50.0
31–90 days	52 (6.7)	25 (7.3)	27 (6.2)	51.9
Unknown or missing value	31 (4.0)	14 (4.1)	17 (3.9)	54.8
Sample condition				
Acceptable	773 (99.4)	341 (99.1)	432 (99.5)	55.9
Unacceptable	1 (0.1)	0 (0)	1 (0.2)	100.0
Unknown or missing value	4 (0.5)	3 (0.9)	1 (0.2)	25.0
Brain sampling method				
Opening skull	594 (76.3)	249 (72.4)	345 (79.5)	58.1
Straw sampling	164 (21.1)	86 (25.0)	78 (18.0)	47.6
Other (Brain sample submitted)	4 (0.5)	4 (1.2)	0 (0)	0.0%
Unknown or missing value	16 (2.1)	5 (1.5)	11 (2.5)	68.8
LFD Lots				
2012	4 (0.5)	0 (0)	4 (100)	100
2102	190 (24.4)	79 (23.0)	111 (25.6)	58.4
2103	197 (25.3)	87 (25.3)	110 (25.3)	55.8
2104	188 (24.2)	97 (28.2)	91 (21.0)	48.4
2201	199 (25.6)	81 (23.5)	118 (27.2)	59.3

^*a*^ Other species include mice, swine, and cattle.

^*b*^ DFAT, direct fluorescent-antibody test; LFD, lateral flow device; ADDRL, animal disease diagnosis and reference laboratory; CAR, cordillera administrative region.

**TABLE 2:** Sensitivity and specificity of the lateral flow device compared to those of the direct fluorescent antibody test^*a*^

	DFAT		Sensitivity (95% CI)	Specificity (95% CI)
	Positive	Negative		
LFD (*n* = 778)				
Positive	418	1	96.3 (94.1–97.9)	99.7 (98.4–100.0)
Negative	16	343		

^*a*^ DFAT, direct fluorescent-antibody test; LFD, lateral flow device; CI, confidence interval.

### Discrepancy results

There were 17 discrepancies in the results between the DFAT and LFD, although we did not perform the confirmatory test using other laboratory methods such as RT-PCR. The results of the analysis of the discrepancy between the DFAT and LFD are presented in Table 3. Only one sample showed a negative DFAT result with a positive LFD result, whereas the other samples showed a positive DFAT result with a negative LFD result. LFD lot no. 2201 had 12 negative LFD and positive DFAT results. Each laboratory had 0–4 discrepancy results, with significantly higher discrepancy rates observed in laboratories that had performed fewer annual rabies tests in the previous years (*P* < 0.05). Most specimens were preserved in an acceptable condition. No significant differences were found in the discrepancy rates based on the species, sample storage duration, sample conditions, and brain sampling methods. While a higher discrepancy rate was observed in LFD lot no. 2201, it should be noted that more kits from this lot were used in laboratories with fewer annual rabies tests in previous years. Logistic regression models showed that the AOR for lot no. 2201 was not significant (AOR 5.16, 95% CI: 0.56–47.67). However, significantly higher AORs were observed in laboratories with fewer yearly rabies tests in previous years (AOR 4.97, 95% CI: 1.49–16.53).

**TABLE 3:** Associations between test characteristics and discrepancy results^*a*^

	No of discrepancy results (Discrepancy rate, %) (*n* = 17/778)	*P* value	Adjusted odds ratio (95% CI)
Laboratory			
ADDRL	1 (0.6)	<0.01	
CAR	1 (2.0)		
I	1 (0.8)		
II	0 (0)		
IV-A	0 (0)		
IV-B	0 (0)		
IV-B_satellite	2 (33.3)		
V	0 (0)		
VI	3 (1.8)		
VII	1 (1.9)		
IX	4 (33.3)		
Davao city (Region XI)	1 (4.8)		
XII	3 (8.3)		
Laboratory groups by yearly rabies tests in the previous years			
Laboratories >200	8 (1.6)	<0.01	Ref
Laboratories 100–200	2 (0.9)		0.49 (0.06–4.34)
Laboratories <100	7 (14.9)		4.97 (1.49–16.53)
Duration between date of sample receive and LFD test (Days)			
Same day	5 (3.2)	0.19	
1–2 days	5 (2.8)		
3–6 days	1 (0.9)		
7–30 days	3 (1.2)		
31–90 days	3 (5.8)		
Brain sampling method			
Opening skull	15 (2.5)	0.69	
Straw sampling	2 (1.2)		
Other (Brain sample submitted)	0 (0)		
Lot no			
2012	1 (25.0)	<0.01	100.07 (3.338–2964.35)
2102	1 (0.5)		Ref
2103	1 (0.5)		0.93 (0.06–15.00)
2104	2 (1.1)		1.84 (0.16–20.79)
2201	12 (6.0)		5.16 (0.56–47.67)

^*a*^ LFD, lateral flow device; CI, confidence interval; ADDRL, animal disease diagnosis and reference laboratory; CAR, cordillera administrative region.

### User experience

The results of the user experience surveys indicate that all users reported LFD to be easier and faster than DFAT (see Table S3). Approximately half of the users perceived the biohazard risk associated with LFD to be lower than that associated with DFAT, whereas the remaining users considered it to be at the same level. Among users, 85% agreed that the LFD could be used as a screening tool, 69% considered it suitable for routine testing, and 38% regarded it as a confirmatory test. The LFD received high recommendations, particularly in cases where there were multiple human bites from the suspected animal or when the animal exhibited signs of rabies.

## DISCUSSION

A multicenter evaluation study conducted in frontline animal diagnostic laboratories in the Philippines demonstrated the high accuracy of the ADTEC LFD, which was supported by a large sample size. Notably, laboratories with low sample processing in previous years exhibited a higher rate of false negative results than other laboratories.

In our literature review, we identified 21 studies that evaluated the diagnostic accuracy of LFD for postmortem rabies diagnosis in animals compared to DFAT (8, 14 – 28, 30 – 34). Among these studies, only 2 had over 200 samples (18, 25), while 11 studies had sample sizes ranging from 100 to 200 (8, 14 – 17, 19, 22, 26, 27, 34). The remaining studies had sample sizes below <100 (20, 21, 24, 26, 28, 30, 31, 33). The largest study included 417 samples, although it used stored samples from three different countries (25). Compared to previous studies, our study had a larger sample size, which enhanced the statistical robustness and increased the generalizability of the findings. Furthermore, the sensitivity in our study was either higher or similarly high compared to the sensitivities reported in other studies. Remarkably, a multicenter study conducted by several international reference laboratories reported inadequate sensitivity for five commercially available LFDs (14). These results were in contrast with the findings of other studies conducted in frontline laboratories (8, 17 – 19, 22, 26). One possible explanation for the lower sensitivity observed in this study is the use of brain specimens with extended storage periods (14, 15, 25, 30). In contrast, studies using fresh or short-storage samples have demonstrated higher sensitivity (8, 15 – 19, 22, 26, 27). Similar findings were observed in our study, in which the majority of samples were fresh or stored for a short period (8, 26). Furthermore, it suggests that there may be variations in the performance of different LFD brands and emphasizes the importance of carefully evaluating and selecting the appropriate LFD for accurate rabies diagnosis. Most of the specimens in this study were in good condition and were well-preserved, making them suitable for the DFAT, although decomposed samples were commonly rejected for rabies testing upon receipt, making their inclusion in our study impossible. The primary contributing factor to the reduced sensitivity of rabies LFD is its lower detection limit compared with DFAT, leading to false-negative results in specimens with low antigen levels (22). Additionally, improper sample preparation is also a potential contributor to lower sensitivity. Inadequate homogenization of the brain tissue can be a potential cause of false negative results. In our study, we identified 17 discrepancies in results (2.2%) between the DFAT and LFD. These discrepancies were more commonly observed in laboratories that had processed fewer rabies samples in the previous years. Among the 17 discrepant results observed between DFAT and LFD, the majority were false negative results, whereas only one false positive result was observed. Although we noticed a higher occurrence of false negative results associated with LFD lot no. 2201 than with other LFD lots, further investigation found that laboratories with fewer rabies samples in previous years used more kits from lot no. 2201. Multivariate logistic regression analysis adjusted with the laboratories categorized based on the number of rabies samples processed in previous years showed no significant association between the specific LFD lots and discrepancy in results. Laboratory VI, which processed over 200 samples in the previous year and used three different lots, showed no significant difference in the discrepancy rate between lot 2201 and the other lots (1.89% vs 1.64%, *P* = 0.91). These findings suggest that laboratories with limited experience in processing rabies samples may be more prone to false negative results when using LFD. This highlights the importance of proper training and quality assessment when implementing LFD. Additionally, further research is needed to explore the potential factors contributing to the observed discrepancies and to identify strategies to minimize false negative results in LFD testing for rabies diagnosis.

Whereas we agree that the DFAT or DRIT should remain the gold standard for testing, the LFD can serve as a valuable tool. Expanding the availability of LFD testing could have several positive effects, including clarifying the actual risk areas for rabies, facilitating prompt responses to outbreaks, and increasing the overall awareness of the disease. These measures can contribute to strengthening rabies surveillance and its effective rapid control, especially in areas where the reference tests, such as the DFAT, are unavailable. At present, the ADTEC LFD is available in the Philippines only for research purposes, though it is relatively expensive at approximately 1,500 Philippine pesos (25 USD) per test. However, when compared to DFAT or PCR, adopting the LFD-based test for the diagnosis of animal rabies is more straightforward, as it requires neither an initial investment nor additional equipment and is highly effective for rapid diagnoses in remote areas where DFAT is unfeasible. It is essential to assess both the diagnostic accuracy and potential challenges of introducing this method to areas lacking prior experience with rabies testing, as highlighted in this study.

Our study had several limitations. First, we did not standardize the DFAT methodology and quality assessments at the participating laboratories, which limited the verification of DFAT accuracy as the reference test. Second, we were unable to confirm the discrepancy between DFAT and LFD using alternative methods, such as RT-PCR, which could have provided additional validation. The participating laboratories were unable to conduct RT-PCR for rabies, and transporting brain specimens with the highly pathogenic rabies virus to a remote central laboratory was challenging. For subsequent studies, researchers might consider transporting used LFD kits for molecular analysis to overcome this limitation (16, 35). Third, a blind assessment of the two tests was not conducted, indicating that the same staff members may have interpreted the results of both DFAT and LFD. This could have introduced bias and potentially affected the agreement between the two tests. Fourth, the variability in the number of test results submitted by the participating laboratories may have influenced the overall findings, particularly because some laboratories had conducted more rabies tests in previous years. This study was not able to evaluate the skills of each technician, such as years of experience. Finally, our study primarily focused on domestic dogs and did not include other wildlife animals. Therefore, the generalizability of our results is limited to endemic areas where domestic dogs are the primary reservoirs. Further research and assessments are necessary to determine the applicability of LFD for the detection of rabies in wild animals.

In conclusion, our study demonstrated the high diagnostic accuracy of LFD for postmortem rabies diagnosis in animals. LFD showed sensitivity and specificity comparable to those of the DFAT reference test. However, certain discrepancies in the results between the LFD and DFAT were observed; these were mainly false negative results. Laboratories with limited experience processing brain specimens may be prone to these discrepancies. Careful training and quality assessment are necessary when introducing LFD. Further research is required to address implementation challenges and optimize LFD utilization in resource-limited settings.

## SUPPLEMENTAL MATERIAL

Supplemental file 1 jcm.00842-23-s0001.pdf Fig. S1 and Tables S1 to S3.

ASM does not own the copyrights to Supplemental Material that may be linked to, or accessed through, an article. The authors have granted ASM a non-exclusive, world-wide license to publish the Supplemental Material files. Please contact the corresponding author directly for reuse.

## References

[1] Miranda MEG , Miranda NLJ . 2020. Rabies prevention in Asia: institutionalizing implementation capacities. Rabies and Rabies Vaccines 103–116. doi:10.1007/978-3-030-21084-7

[2] Bourhy H , Dautry-Varsat A , Hotez PJ , Salomon J . 2010. Rabies, still neglected after 125 years of vaccination. PLoS Negl Trop Dis 4:e839. doi:10.1371/journal.pntd.0000839 21152052 PMC2994912

[3] Hampson K , Coudeville L , Lembo T , Sambo M , Kieffer A , Attlan M , Barrat J , Blanton JD , Briggs DJ , Cleaveland S , et al. . 2015. Estimating the global burden of Endemic canine Rabies. PLoS Negl Trop Dis 9:e0003786. doi:10.1371/journal.pntd.0003786 25881058 PMC4400070

[4] Nyasulu PS , Weyer J , Tschopp R , Mihret A , Aseffa A , Nuvor SV , Tamuzi JL , Nyakarahuka L , Helegbe GK , Ntinginya NE , Gebreyesus MT , Doumbia S , Busse R , Drosten C . 2021. Rabies mortality and morbidity associated with animal bites in Africa: a case for integrated rabies disease surveillance, prevention and control: a Scoping review. BMJ Open 11:e048551. doi:10.1136/bmjopen-2020-048551 PMC8640643 34857556

[5] Hampson K , Dushoff J , Cleaveland S , Haydon DT , Kaare M , Packer C , Dobson A . 2009. Transmission dynamics and prospects for the elimination of canine rabies. PLoS Biol 7:e53. doi:10.1371/journal.pbio.1000053 19278295 PMC2653555

[6] Franka R , Wallace R . 2018. Rabies diagnosis and surveillance in animals in the era of rabies elimination. Rev Sci Tech 37:359–370. doi:10.20506/rst.37.2.2807 30747142

[7] World Health Organization . 2018. WHO expert consultation on rabies: third report. World Health Organization, Geneva. Available from: https://apps.who.int/iris/handle/10665/272364

[8] Kimitsuki K , Saito N , Yamada K , Park C-H , Inoue S , Suzuki M , Saito-Obata M , Kamiya Y , Manalo DL , Demetria CS , Mananggit MR , Quiambao BP , Nishizono A . 2020. Evaluation of the diagnostic accuracy of lateral flow devices as a tool to diagnose rabies in post-mortem animals. PLoS Negl Trop Dis 14:e0008844. doi:10.1371/journal.pntd.0008844 33151941 PMC7671516

[9] Rupprecht CE , Fooks AR , Abela-Ridder B , World Health Organization . 2018. Laboratory techniques in Rabies. 5th ed. Vol. 1. World Health Organization, Geneva. https://apps.who.int/iris/handle/10665/310836.

[10] World Organization for Animal Health Manual of Diagnostic Tests and Vaccines for Terrestrial Animals . 2023. Chapter 3.1.18, Rabies (infection with Rabies virus and other Lyssaviruses) (version adopted in May 2023). In World Organization for animal health. Manual of diagnostic tests and vaccines for terrestrial animals

[11] Patrick EM , Bjorklund BM , Kirby JD , Nelson KM , Chipman RB , Rupprecht CE . 2019. Enhanced rabies surveillance using a direct rapid immunohistochemical test. J Vis Exp. doi:10.3791/59416 31107436

[12] Dürr S , Naïssengar S , Mindekem R , Diguimbye C , Niezgoda M , Kuzmin I , Rupprecht CE , Zinsstag J . 2008. Rabies diagnosis for developing countries. PLoS Negl Trop Dis 2:e206. doi:10.1371/journal.pntd.0000206 18365035 PMC2268742

[13] Wadhwa A , Wilkins K , Gao J , Condori Condori RE , Gigante CM , Zhao H , Ma X , Ellison JA , Greenberg L , Velasco-Villa A , Orciari L , Li Y . 2017. A pan-lyssavirus taqman real-time RT-PCR assay for the detection of highly variable rabies virus and other lyssaviruses. PLoS Negl Trop Dis 11:e0005258. doi:10.1371/journal.pntd.0005258 28081126 PMC5230753

[14] Klein A , Fahrion A , Finke S , Eyngor M , Novak S , Yakobson B , Ngoepe E , Phahladira B , Sabeta C , De Benedictis P , Gourlaouen M , Orciari LA , Yager PA , Gigante CM , Knowles MK , Fehlner-Gardiner C , Servat A , Cliquet F , Marston D , McElhinney LM , Johnson T , Fooks AR , Müller T , Freuling CM . 2020. Further evidence of inadequate quality in lateral flow devices commercially offered for the diagnosis of rabies. Trop Med Infect Dis 5:13. doi:10.3390/tropicalmed5010013 31963635 PMC7157750

[15] Servat A , Picard-Meyer E , Robardet E , Muzniece Z , Must K , Cliquet F . 2012. Evaluation of a rapid immunochromatographic diagnostic test for the detection of Rabies from brain material of European mammals. Biologicals 40:61–66. doi:10.1016/j.biologicals.2011.12.011 22245544

[16] Mauti S , Léchenne M , Naïssengar S , Traoré A , Kallo V , Kouakou C , Couacy-Hymann E , Gourlaouen M , Mbilo C , Pyana PP , Madaye E , Dicko I , Cozette P , De Benedictis P , Bourhy H , Zinsstag J , Dacheux L . 2020. Field postmortem rabies rapid immunochromatographic diagnostic test for resource-limited settings with further molecular applications. J Vis Exp 160. doi:10.3791/60008 32658185

[17] Tenzin T , Lhamo K , Rai PB , Tshering D , Jamtsho P , Namgyal J , Wangdi T , Letho S , Rai T , Jamtsho S , Dorji C , Rinchen S , Lungten L , Wangmo K , Lungten L , Wangchuk P , Gempo T , Jigme K , Phuntshok K , Tenzinla T , Gurung RB , Dukpa K . 2020. Evaluation of a rapid immunochromatographic test kit to the gold standard fluorescent antibody test for diagnosis of rabies in animals in Bhutan. BMC Vet Res 16:183. doi:10.1186/s12917-020-02405-4 32513172 PMC7281917

[18] Yale G , Gibson AD , Mani RS , P K H , Costa NC , Corfmat J , Otter I , Otter N , Handel IG , Bronsvoort BM , Mellanby RJ , Desai S , Naik V , Gamble L , Mazeri S . 2019. Evaluation of an immunochromatographic assay as a canine rabies surveillance tool in Goa, India. Viruses 11:649. doi:10.3390/v11070649 31311178 PMC6669590

[19] Léchenne M , Naïssengar K , Lepelletier A , Alfaroukh IO , Bourhy H , Zinsstag J , Dacheux L . 2016. Validation of a rapid rabies diagnostic tool for field surveillance in developing countries. PLoS Negl Trop Dis 10:e0005010. doi:10.1371/journal.pntd.0005010 27706156 PMC5051951

[20] Sharma P , Singh CK , Narang D . 2015. Comparison of immunochromatographic diagnostic test with Hheminested reverse transcriptase polymerase chain reaction for detection of rabies virus from brain samples of various species. Vet World 8:135–138. doi:10.14202/vetworld.2015.135-138 27047061 PMC4774692

[21] Voehl KM , Saturday GA . 2014. Evaluation of a rapid immunodiagnostic rabies field surveillance test on samples collected from military operations in Africa, Europe, and the middle East. US Army Med Dep J:27–32. 25074599

[22] Reta T , Teshale S , Deresa A , Ali A , Getahun G , Baumann MPO , Muller T , Freuling CM . 2013. Evaluation of rapid Immunodiagnostic test for Rabies diagnosis using clinical brain samples in Ethiopia. J Vet Sci Med Diagn. doi:10.4172/2325-9590.1000117

[23] Yang D-K , Shin E-K , Oh Y-I , Lee K-W , Lee C-S , Kim S-Y , Lee J-A , Song J-Y . 2012. Comparison of four diagnostic methods for detecting rabies viruses circulating in Korea. J Vet Sci 13:43–48. doi:10.4142/jvs.2012.13.1.43 22437535 PMC3317456

[24] Nishizono A , Khawplod P , Ahmed K , Goto K , Shiota S , Mifune K , Yasui T , Takayama K , Kobayashi Y , Mannen K , Tepsumethanon V , Mitmoonpitak C , Inoue S , Morimoto K . 2008. A simple and rapid immunochromatographic test kit for rabies diagnosis. Microbiol Immunol 52:243–249. doi:10.1111/j.1348-0421.2008.00031.x 18426399 PMC7168491

[25] Ahmed K , Wimalaratne O , Dahal N , Khawplod P , Nanayakkara S , Rinzin K , Perera D , Karunanayake D , Matsumoto T , Nishizono A . 2012. Evaluation of a monoclonal antibody-based rapid Immunochromatographic test for direct detection of rabies virus in the brain of humans and animals. Am J Trop Med Hyg 86:736–740. doi:10.4269/ajtmh.2012.11-0332 22492163 PMC3403755

[26] Mananggit MR , Manalo DL , Saito N , Kimitsuki K , Garcia AMG , Lacanilao PMT , Ongtangco JT , Velasco CR , Del Rosario MVA , Lagayan MGO , Yamada K , Park C-H , Inoue S , Suzuki M , Saito-Obata M , Kamiya Y , Demetria CS , Quiambao BP , Nishizono A . 2021. Lateral flow devices for samples collected by straw sampling method for postmortem canine rabies diagnosis. PLoS Negl Trop Dis 15:e0009891. doi:10.1371/journal.pntd.0009891 34882672 PMC8659307

[27] Gury Dohmen F , Kovacs E , Prestrera NE , Beltrán FJ . 2018. Evaluation of a rapid immunochromatographic diagnostic test (RIDT) for diagnosis of rabies in samples from Argentina. J Infect Dev Ctries 12:415–421. doi:10.3855/jidc.9552 31940292

[28] Kang B , Oh J , Lee C , Park B-K , Park Y , Hong K , Lee K , Cho B , Song D . 2007. Evaluation of a rapid immunodiagnostic test kit for rabies virus. J Virol Methods 145:30–36. doi:10.1016/j.jviromet.2007.05.005 17628707

[29] National Rabies prevention and control program in the Philippines. manula of pcocedures. 2019. Available from: https://doh.gov.ph/sites/default/files/publications/Rabies%20Manual_MOP_2019%20nov28.pdf

[30] Eggerbauer E , de Benedictis P , Hoffmann B , Mettenleiter TC , Schlottau K , Ngoepe EC , Sabeta CT , Freuling CM , Müller T . 2016. Evaluation of six commercially available rapid immunochromatographic tests for the diagnosis of rabies in brain material. PLoS Negl Trop Dis 10:e0004776. doi:10.1371/journal.pntd.0004776 27336943 PMC4918935

[31] Certoma A , Lunt RA , Vosloo W , Smith I , Colling A , Williams DT , Tran T , Blacksell SD . 2018. Assessment of a rabies virus rapid diagnostic test for the detection of Australian bat lyssavirus. Trop Med Infect Dis 3:109. doi:10.3390/tropicalmed3040109 30287778 PMC6306826

[32] Naïssengar K , Oussiguere A , Madaye E , Mbaipago N , Mindekem R , Moyengar R , Madjadinan A , Ngandolo R , Zinsstag J , Léchenne M . 2021. Challenges to improved animal rabies surveillance: experiences from pilot implementation of decentralized diagnostic units in chad. Acta Trop 221:105984. doi:10.1016/j.actatropica.2021.105984 34058158

[33] Ahmad A , Singh CK . 2016. Comparison of rapid immunodiagnosis assay kit with molecular and Immunopathological approaches for diagnosis of rabies in cattle. Vet World 9:107–112. doi:10.14202/vetworld.2016.107-112 27051193 PMC4819342

[34] Su MPA . 2015. Evaluation of two rapid diagnostic tests for rabies diagnosis under field and laboratory conditions in Nigeria. Journal of Vaccines & Vaccination 06

[35] Mauhay JD , Saito N , Kimitsuki K , Mananggit MR , Cruz JL , Lagayan MG , Garcia AM , Lacanilao PM , Yamada K , Saito-Obata M , Manalo DL , Demetria CS , Quiambao BP , Nishizono A . 2023. Molecular analysis of rabies virus using RNA extracted from used lateral flow devices. J Clin Microbiol 61:e0154322. doi:10.1128/jcm.01543-22 36840574 PMC10035306

