# Supplementary material for: Evaluation of lateral flow devices for postmortem rabies diagnosis in animals in the Philippines: a multicenter study
Source: J Clin Microbiol. 2023 Nov 22;61(12):e00842-23. doi: 10.1128/jcm.00842-23 (PMC10729751; doi:10.1128/jcm.00842-23)
Supplement: Supplemental file 1 — Fig. S1 and Tables S1 to S3. [file jcm.00842-23-s0001.pdf]

Supplementary Table S1.

Results of Baseline Survey: Laboratory Characteristics, Test Parameters, and Rabies Diagnostic Practices among Participating Laboratories

Laboratory	ADDRL	CAR	I	II	IV-A	IV-B	IV-B_sat	V	VI	VII	IX	Davao	XII
Average of yearly positive samples of DFAT based on the number of 2019 and 2022	66	29	93	50	36	6	4	74	120	61	20	28	82
Average of yearly total samples of DFAT	235	122	230	108	101	29	23	181	206	193	48	92	262
Positivity rate of DFAT	27.9%	23.5%	40.2%	46.0%	35.6%	19.3%	17.8%	40.6%	58.2%	31.6%	41.1%	30.6%	31.3%
No. of Veterinarians who perform rabies test in the laboratory	1	4	3	5	5	2	2	3	2	2	2	1	4
No. of non-Veterinarians who perform rabies test in the laboratory	0	1	0	0	0	2	1	2	1	1	1	3	1
Turn-around time of rabies tests? (from the sample arrival to the release of the result)? (Hours)	4	72	24	24	36	5	4	51	6	72	8	8	2
Turn-around time of DFAT (from the sample collection until the reading of slides)? (Hours)	2	3	2	6	2	3.5	3	18	4	6	2	2	2
Previous experience of LFD	No	No	No	No	Yes	Yes	No	No	Yes	No	No	No	No
Previous experience of the occipital foramen route brain sampling (Simplified sampling method)	No	No	Yes	No	No	Yes	No	No	No	No	No	No	No
Previous experience of problems resulting to the non-performance of DFAT within the last two years.	Yes	Yes	No	No	No	Yes	Yes	No	No	No	No	No	No

DFAT, Direct Fluorescent-Antibody Test; LFD, Lateral Flow Device; CI, confidence interval

ADDRL, Animal Disease Diagnosis and Reference Laboratory; CAR, Cordillera Administrative Region

Supplementary Figure S1. Examples of LDF results.

(A) Negative results showing only control line. (B) Positive result showing both control and test lines

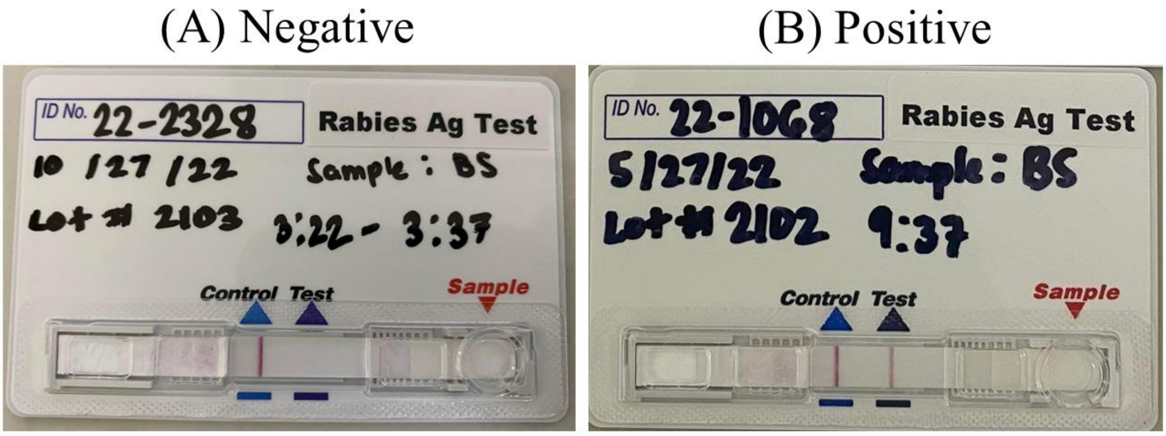

Supplementary Table S2. STARD (Standards for Reporting of Diagnostic Accuracy) checklist and diagram

Title "Evaluation of lateral flow devices for post-mortem rabies diagnosis in animals in the Philippines: A multicenter study"

Section & Topic	No	Item	Reported on page #
**TITLE OR ABSTRACT**			
	**1**	Identification as a study of diagnostic accuracy using at least one measure of accuracy (such as sensitivity, specificity, predictive values, or AUC)	Page 3, Abstract
**ABSTRACT**			
	**2**	Structured summary of study design, methods, results, and conclusions (for specific guidance, see STARD for Abstracts)	Abstract
**INTRODUCTION**			
	**3**	Scientific and clinical background, including the intended use and clinical role of the index test	Page 3- 5
	**4**	Study objectives and hypotheses	Page 5
**METHODS**			
Study design	**5**	Whether data collection was planned before the index test and reference standard were performed (prospective study) or after (retrospective study)	Page 6-8
Participants	**6**	Eligibility criteria	Page 6
	**7**	On what basis potentially eligible participants were identified (such as symptoms, results from previous tests, inclusion in registry)	Page 6
	**8**	Where and when potentially eligible participants were identified (setting, location and dates)	Page 6, Figure 1
	**9**	Whether participants formed a consecutive, random or convenience series	Page 7
Test methods	**10a**	Index test, in sufficient detail to allow replication	Page 7-8, Reference 11 & 24
	**10b**	Reference standard, in sufficient detail to allow replication	Reference 28
	**11**	Rationale for choosing the reference standard (if alternatives exist)	NA
	**12a**	Definition of and rationale for test positivity cut-offs or result categories of the index test, distinguishing pre-specified from exploratory	Reference 11 & 24
	**12b**	Definition of and rationale for test positivity cut-offs or result categories of the reference standard, distinguishing pre-specified from exploratory	Reference 11 & 24
	**13a**	Whether clinical information and reference standard results were available to the performers/readers of the index test	Not performed
	**13b**	Whether clinical information and index test results were available to the assessors of the reference standard	Not performed
Analysis	**14**	Methods for estimating or comparing measures of diagnostic accuracy	Page 8-9
	**15**	How indeterminate index test or reference standard results were handled	Page 10, 12
	**16**	How missing data on the index test and reference standard were handled	Page 9, STARD diagram
	**17**	Any analyses of variability in diagnostic accuracy, distinguishing pre-specified from exploratory	NA

	**18**	Intended sample size and how it was determined	NA
**RESULTS**			
Participants	**19**	Flow of participants, using a diagram	STARD diagram
	**20**	Baseline demographic and clinical characteristics of participants	Table 1
	**21a**	Distribution of severity of disease in those with the target condition	NA
	**21b**	Distribution of alternative diagnoses in those without the target condition	NA
	**22**	Time interval and any clinical interventions between index test and reference standard	NA
Test results	**23**	Cross tabulation of the index test results (or their distribution) by the results of the reference standard	Table 2
	**24**	Estimates of diagnostic accuracy and their precision (such as 95% confidence intervals)	Table 2
	**25**	Any adverse events from performing the index test or the reference standard	NA
**DISCUSSION**			
	**26**	Study limitations, including sources of potential bias, statistical uncertainty, and generalisability	Page 13
	**27**	Implications for practice, including the intended use and clinical role of the index test	Page 10 & 13
**OTHER INFORMATION**			
	**28**	Registration number and name of registry	NA
	**29**	Where the full study protocol can be accessed	Reference 11 & 24
	**30**	Sources of funding and other support; role of funders	Page 18

Abbreviation: NA, not applicable; LFD, lateral flow device; DFAT, direct fluorescent antibody test

STARD Diagram

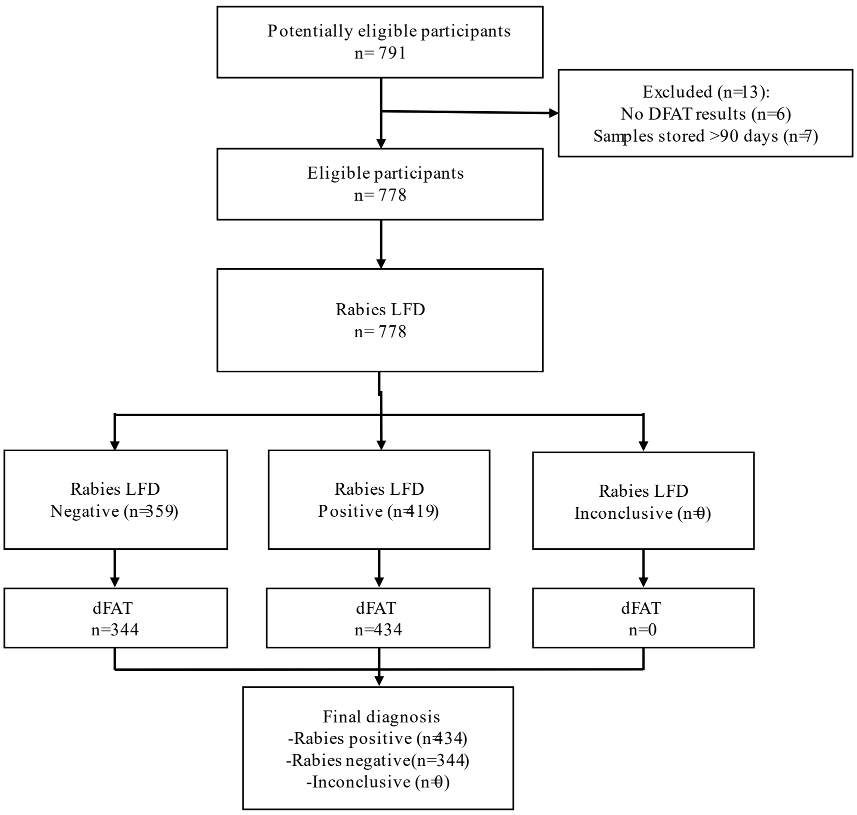

Supplementary Table S3: Resuls of user experinece survey

		N	%		Comments
Easiness of test procedures of LFD	Very easy	7	54%	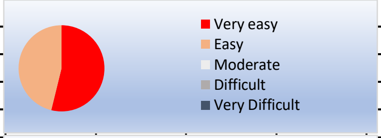	(Very easy) The procedures are very easy to do.<br>We did not encounter any problem. Even new lab technician also could do it easily.<br>(Easy) The procedures are easy and fast.
	Easy	6	46%		
	Moderate	0	0%		
	Difficult	0	0%		
	Very Difficult	0	0%		
Easiness of test procedures of LFD compared to those of DFAT	Much easier than DFAT	5	38%	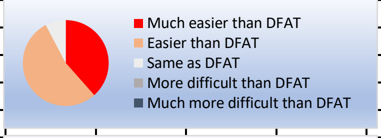	(Much easier than DFAT) It is much easier to use than DFAT andWe can have the result immediately.<br>(same as DFAT) In the lab, I only do the reading of DFAT so for me it is the same. But if you do DFAT from the start, LFD is easier to do.
	Easier than DFAT	7	54%		
	Same as DFAT	1	8%		
	More difficult than DFAT	0	0%		
	Much more difficult than DFAT	0	0%		
Turnaround time of the LFD procedure and test results	Very Rapid	3	23%	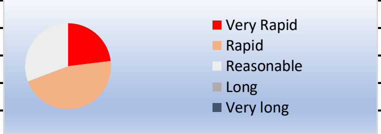	(Very rapid) The duration of reading the results of LFD is very rapid.<br>(Rapid) if the sample is positive, you only need to wait for a few minutes before the test line will appear.<br>You only need to wait for 15 minutes in negative samples to ensure that they are truly negative.<br>(Rasonable) The result can also be read earlier if the result is very positive.
	Rapid	6	46%		
	Reasonable	4	31%		
	Long	0	0%		
	Very long	0	0%		
Turnaround time of LFD compared to those of DFAT	Much Faster than DFAT	7	54%	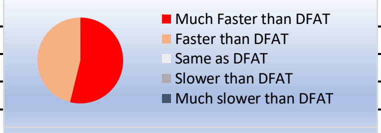	(Much Faster than DFAT) It is substantially faster than DFAT.
	Faster than DFAT	6	46%		
	Same as DFAT	0	0%		
	Slower than DFAT	0	0%		
	Much slower than DFAT	0	0%		
Interpretation of the results of LFD	Very easy	6	46%	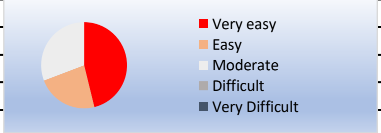	(Very easy) However, some positive samples only produces a faint line.<br>(Moderate) Sometime we observed faint lines. If the line is weak, it is slightly hard to interpret.
	Easy	3	23%		
	Moderate	4	31%		
	Difficult	0	0%		
	Very Difficult	0	0%		
Interpretation of the results of LFD compared to those of DFAT	Much clearer than DFA	2	15%	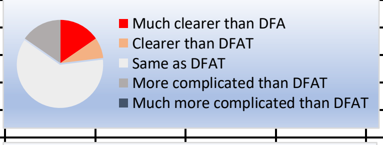	(Same as DFAT) In LFD, we cab only read a line.<br>It is the same as reading DFAT results. However, a negative result in LFD might be a false negative.<br>(More difficult) It still depends on the color and intensity of the test line. To the untrained eye, it is very hard. But with time and experience, it somewhat became easier.
	Clearer than DFAT	1	8%		
	Same as DFAT	8	62%		
	More complicated than DFAT	2	15%		
	Much more complicated than DFA	0	0%		
Biohazard risk during the LFD test	Very low	0	0%	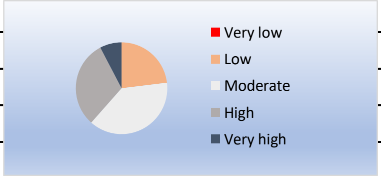	(Low) Biohazard risk is low especially if the test kit is used in tandem with Straw Method Collection because there is no craniotomy involved. If the analyst also has complete PPE, it is not very hazardous.<br>(Moderate) There is still biohazard risk. But, using the test kit with straw method collection is lower baiohazrd risk compared to craniotomy.<br>(Very high) -need proper equipments and PPE.<br>(High) - Using the LFD kit is hazardous if the lab tech is not well-trained. The safety requirements(vaccine requirement of the lab tech, type of room, and PPE) should be necessary.<br>-Caution should be taken if there is sample leakage from the tubes.
	Low	3	23%		
	Moderate	5	38%		
	High	4	31%		
	Very high	1	8%		
Biohazard risk of LFD compared to DFAT	Much lower than DFAT	0	0%	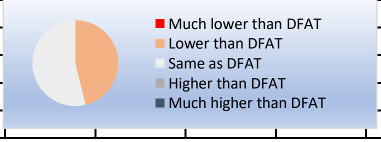	(Lower) It is lower because there is lesser time of exposure from the brains sample.<br>(Same) It still the same as DFAT because we are still exposed to the brain sample.<br>Craniotomy or Straw Method Collection, we are still exposing yourself to the virus.<br>Because we both do them inside a biosafety cabinet.
	Lower than DFAT	6	46%		
	Same as DFAT	7	54%		
	Higher than DFAT	0	0%		
	Much higher than DFAT	0	0%		

Cont. Resuls of user experinece survey

		N	%		Comments
Recommendation to use LFD kits as a screening test for rabies diagnosis?	Strongly Agree	4	31%	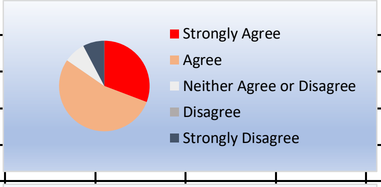	(Strongly agree) - We recommend LFD kits for screening rabies samples.<br>(Agree) - Although DFAT is still better for rabies diagnosis, it can be used as a screening test<br>(Neither agree or disagree) If there is screening test, it cause double the amount of our work so its better to proceed with DFAT immediately.<br>Particularly, LGUs can use it so they can easily screen the sample and lower the burden of samples coming to our laboratory.<br>(Strongly disagree) The sensitivity of the kit is still only 94%. There is a possibility that they might not detect the rabies in a sample if the viral load is low.
	Agree	7	54%		
	Neither Agree or Disagree	1	8%		
	Disagree	0	0%		
	Strongly Disagree	1	8%		
Recommendation to use the LFD kit for routine diagnosis at regional government animal laboartory	Strongly Agree	5	38%	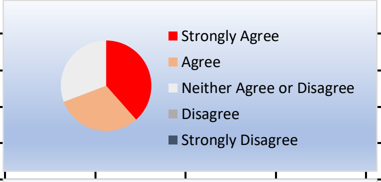	(Strongly agree) LFD test is easy and fast. The sensitivity is also high. - Reading DFAT relus on the skills of analyst. But LFD is much easier and can show clear results.<br>(Agree) It can be used as part of routine diagnosis in the laboratory as it is comparable to DFAT.<br>(Neither Agree or Disagree) - we can use the kit in our lab if the OIE will approve and recommend the use of this lateral flow devices for rabies diagnosis.<br>DFAT is still the better choice for confirmation.- DFAT is still the gold standard<br>This kit is very easy to use as a screening test but it can be an additional expenses for us. But if we have adequate budget, we can use it for our rabies routine diagnosis.
	Agree	4	31%		
	Neither Agree or Disagree	4	31%		
	Disagree	0	0%		
	Strongly Disagree	0	0%		
Recommendation to use LFD kits as a confirmatory test for rabies diagnosis	Strongly Agree	2	15%	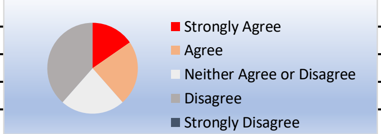	(Neither agree or disagree) -As RADDL, we still need to follow a standard protocol from BAI. Also, DFAT is still the gold standard for rabies diagnosis.<br>(Disagree) Confirmatory test should still be DFAT because it is the gold standard for rabies diagnosis.<br>Because ofthe false negative by LFD, we still prefer DFAT as our confirmatory test.<br>Based on our previous experiences, we encountered false negative samples.<br>(Strongly disagree) -DFAT is still the confirmatory test.
	Agree	3	23%		
	Neither Agree or Disagree	3	23%		
	Disagree	5	38%		
	Strongly Disagree	0	0%		
Recommendation to purchage LFD kits to central government office (Department of Agriculture) purchase a kit?	Strongly Agree	2	15%	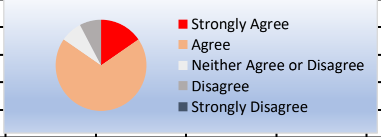	(Strongly agree) - the kit is useful for screening.<br>(Agree) It is beneficial for screening purposes. If the test is positive, control measures can be taken immediately. It is beneficial, If a laboratory needs immediate results and no analyst who can do DFAT is available.We recommend that BAI should purchase the kits.<br>(Neither agree or disagree) - If used as a screening tests, we are not sure if our LGU will comply with our request. Inside the lab, we prefer to use DFAT directly.
	Agree	9	69%		
	Neither Agree or Disagree	1	8%		
	Disagree	1	8%		
	Strongly Disagree	0	0%		
Recommendation to purchage LFD kits to regional government office purchase the kit	Strongly Agree	2	15%	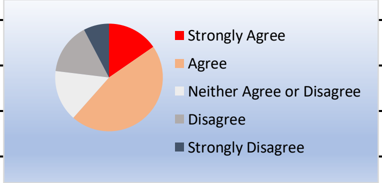	(Strongly agree) - The kit is useful for screening.<br>(Agree) - if we have budget for it. If DA cannot provide the kits, our office can buy and we can ask clients to pay a fee. - When DFAT test is not available, LFD can be used. .<br>(Disagree) - Because it might be a burden to our budget and all samples we receive are required to undergo DFAT<br>-It can be additional expenses for us so it is better to test the samples using DFAT. Although the procedures are easy to do, it is a hassle that everytime that the result in the kit is negative we still need to test it using DFAT. Also, the LFD result cannot be released to the clients but if the BAI approved of this, we can use it then.<br>(Strongly disagree) - If used as a screening tests, we are not sure if our LGU will comply with our request. Inside the lab, we prefer to use DFAT directly.
	Agree	6	46%		
	Neither Agree or Disagree	2	15%		
	Disagree	2	15%		
	Strongly Disagree	1	8%		
Recommendation to use LFDs by local government units such as city veterinary office and municipal veterinaly office	Strongly Agree	4	31%	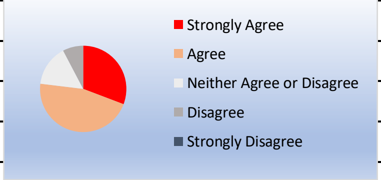	(Strongly agree) For early detection of rabies and immediate action can be taken. This is very helpful in islands very far from our laboratoryLFD lab is easy to establish compared with DAT. Local municipalities that are very far from our laboratory can use the LFD kits especially if they need to release results urgently.<br>(Agree) Agree if the criteria for LFD are finalized.<br>They can use it for screening purposes and actions can be taken immediately for positive samples.<br>However, DFAT is still needed for confirmation. LGUs can directly report the results to the owners/victims.<br>(Neither agree or disagree) We will still advise them to bring the sample immediately to RADDL if the LFD result is negative.<br>(Disagree) To perform LFD, it should be done at BSL-2 laboratory. But if they have an established laboratory, they can perform LFDs.
	Agree	6	46%		
	Neither Agree or Disagree	2	15%		
	Disagree	1	8%		
	Strongly Disagree	0	0%		
Recommendation to use LFDs by private animal clinic	Strongly Agree	4	31%	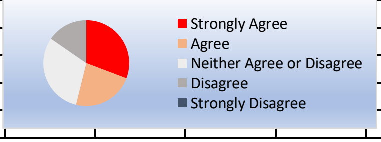	(Strongly agree) For early detection because we have previous encounters with their referrals and they are mostly positive. Samples can still be brought to RADDL as well as their reports.<br>(Agree) This is for screening purposes and so that immediate action can be taken. However, they still need to submit the sample to RADDL for DFAT.<br>(Disagree) To perform LFD should be done at a BSL-2 laboratory.<br>This might incur additional fees for the clients unlike in RADDLs .
	Agree	3	23%		
	Neither Agree or Disagree	4	31%		
	Disagree	2	15%		
	Strongly Disagree	0	0%		

Cont. Resuls of user experinece survey

What situations would you suggest using the LFD kit?

		N	%		Comments
Animal is involved in a multiple unprovoked biting incident/s	Strongly Agree	9	69%	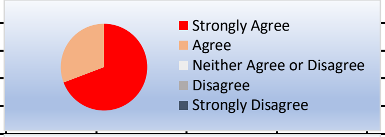	(Strongly agree) We commonly encounter this situation. Most cases were test positive. LFD can show rapid results.
	Agree	4	31%		
	Neither Agree or Disagree	0	0%		
	Disagree	0	0%		
	Strongly Disagree	0	0%		
Animals showing rabies signs	Strongly Agree	9	69%	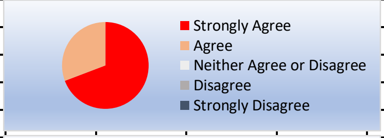	(Strongly agree) We often encounter this situation. Most cases were test positive. LFD can show rapid results.<br>As long as there visible rabies signs, it is okay to test using the kit.
	Agree	4	31%		
	Neither Agree or Disagree	0	0%		
	Disagree	0	0%		
	Strongly Disagree	0	0%		
Sudden death with unknown reson	Strongly Agree	4	31%	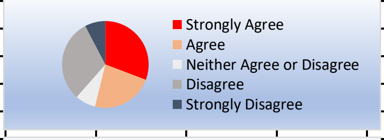	(Strongly agree) Unknown and sudden death must be due to rabies even if there is no history provided.<br>(Agree) It might not be rabid but if the client wants to have immediate results, we can use LFD. Yes. But we still need DFAT after if LFD test is negative.<br>(Disagree) DFAT should be preferred in this situation. It is better to test directly using DFAT.<br>(Strongly disagree) The sensitivity of LFD in this situation might not be good due to low viral load in the sample.
	Agree	3	23%		
	Neither Agree or Disagree	1	8%		
	Disagree	4	31%		
	Strongly Disagree	1	8%		
Stray animals found dead	Strongly Agree	4	31%	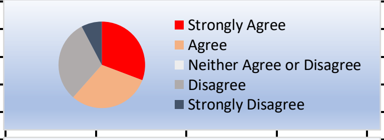	(Agree) Because it is highly suspected with rabies if it is stray dog. It might be positive.<br>(Disagree) It is better to test directly using DFAT so it is less expensive.<br>(Strongly disagree) The sensitivity of the sample to the kit might not be good
	Agree	4	31%		
	Neither Agree or Disagree	0	0%		
	Disagree	4	31%		
	Strongly Disagree	1	8%		
Animal roadkill	Strongly Agree	2	15%	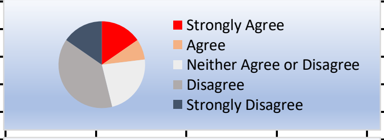	(Strongly agree) - We have previously incidents with this kind of situation and they are mostly positive.<br>(Neither agree or disagree) We are not sure about the history of the animal and the cause of death so I would not suggest this. - It has lesser chances of being rabies positive.<br>(Strongly disagree) - The sensitivity of the sample to the kit might not be good due to low viral load in the sample. The brain may also be decomposing and this might affect the result.
	Agree	1	8%		
	Neither Agree or Disagree	3	23%		
	Disagree	5	38%		
	Strongly Disagree	2	15%		
Samples from dog catching activity (stray dogs)	Strongly Agree	2	15%	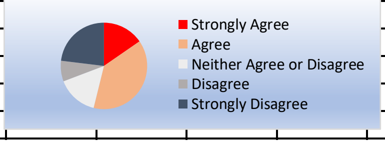	(Agree) -Most samples from surveillance are negative but if they have available supplies of the kit. - If the LGU agree using the kits.<br>(Disagree) Based on our previous experiences with dogs submitted by impounding, they are mostly negative.<br>It is better to test directly using DFAT because DFAT is less expensive.<br>(Strongly disagree) The sensitivity of the sample to the kit might not be good
	Agree	5	38%		
	Neither Agree or Disagree	2	15%		
	Disagree	1	8%		
	Strongly Disagree	3	23%		
